# Primary pleural schwannoma: a rare case report

**DOI:** 10.25122/jml-2024-0308

**Published:** 2024-10

**Authors:** Moaaz Amir, Salwa Sheikh

**Affiliations:** 1 Department of Medicine, Imam Abdulrahman Bin Faisal University, Dammam, Saudi Arabia; 2 Johns Hopkins Aramco Healthcare, Department of Pathology, Dhahran, Saudi Arabia

**Keywords:** schwannoma, pleural schwannoma, benign peripheral nerve sheath tumor

## Abstract

Schwannomas are peripheral nerve sheath tumors that rarely arise from autonomic nerves of the pleural lining. Most often, they present as slow-growing tumors and are asymptomatic. Herein, we describe the case of an elderly male patient who presented with severe chest pain. An initial chest X-ray detected a suspicious lung lesion. Further examination with a positron emission tomography-computed tomography (PET-CT) scan revealed a hypermetabolic mass in the base of the left pleura. A core needle biopsy of the lesion showed features consistent with a benign pleural schwannoma on histopathological assessment. It is crucial for clinicians and radiologists to recognize this unusual presentation to properly diagnose and appropriately treat patients with this type of tumor, as malignant lesions—whether primary or metastatic—are always part of the differential diagnosis in such cases.

## INTRODUCTION

Primary pleural schwannomas (neurilemmoma) are peripheral nerve sheath tumors that arise from the autonomic nerve sheaths within the pleura, which is an exceedingly rare location for this tumor [1]. Most of these tumors present as slow-growing benign neoplasms that have a male predilection [2]. We herein report a case of an elderly male patient who presented to the emergency department with severe retrosternal pain and a suspicious lesion on the initial chest X-ray. Further evaluation using PET-CT revealed a hypermetabolic left pleural-based mass, which, upon histopathologic examination, revealed findings consistent with benign pleural schwannoma. Clinicians and radiologists must be aware of this uncommon manifestation to appropriately manage such patients, as the differential diagnosis would include malignancy in this age and location.

## CASE PRESENTATION

**Figure 1:**
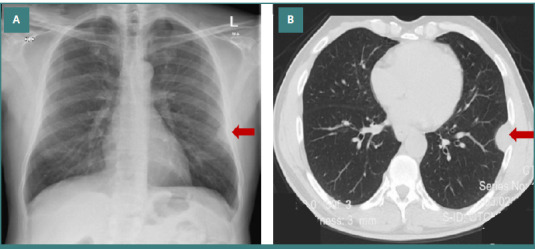
Radiological image of pleural mass. A, Plain chest X-ray showing a left pleural-based mass. B, CT scan highlighting the pleural-based elliptical mass

This is a case of a 63-year-old man who presented to the emergency department complaining of severe retrosternal burning sensation and pain. He had multiple medical problems, including bronchial asthma, hyperlipidemia, benign prostatic hypertrophy, and chronic prostatitis. Physical examination was unremarkable. Chest X-ray showed a well-defined 3 cm in size left pleural-based soft tissue mass close to the left seventh rib. No lung infiltrate, cardiomegaly, or pleural effusion was present (Figure 1AB). The patient was a smoker for 35 years and quit only a few years ago. He had since retired from an office-based job with no history of exposure to chemicals or carcinogens, including asbestosis.

The patient was given symptomatic treatment and was discharged home with an appointment for a chest CT scan for further workup of the pleural-based mass. An unenhanced CT scan of the chest showed a 3.2 cm x 2.1 cm x 2.9 cm well-defined elliptical-shaped left lower lobe pleural-based mass. The remaining pleural surfaces were unremarkable. No lung lesions were identified. The patient was referred to pulmonology. A PET-CT scan showed a hypermetabolic pleural mass with no evidence of any other hypermetabolic suspicious lesion in the study.

**Figure 2:**
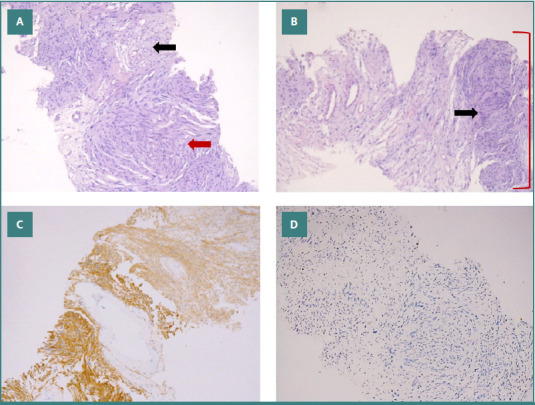
Pathological image of biopsy. A, Sections showing spindle cell proliferation with hypercellular areas (red arrow) alternating with hypocellular areas (black arrow) (10x). B, Sections displaying Verocay bodies formed by palisading of spindled neoplastic nuclei (black arrow) in hypercellular areas (red bracket) (10x). C, Neoplastic spindle cells strongly positive for S-100 and CD56 (10x). D, Spindle cells negative for Cytokeratin, CD34, desmin, and CK7 (10x).

A CT-guided biopsy was performed by interventional radiology. Histopathologic examination of the needle core biopsies revealed a lesion composed of proliferating elongated spindle cells with eosinophilic cytoplasm and bland nuclei (Figure 2A-D). There was no evidence of cytologic atypia, increased mitotic activity, or necrosis. The spindle cells exhibited areas of hypercellularity and nuclear palisading/Verocay bodies (Antoni Type A) alternating with hypocellular, somewhat edematous foci (Antoni Type B). The spindle cells were strongly positive for S100 and CD56. The cells were negative for cytokeratin, desmin, CD34, and CK7. Ki-67 showed a low proliferation index, with less than 1% of the cells being positive. The findings were characteristic of benign schwannoma.

## DISCUSSION

Schwannomas are benign peripheral nerve sheath tumors that commonly involve the flexor surfaces of the extremities, with a predilection for the upper limbs. They are also frequently found in the head and neck region, including the cerebellopontine angle, oral cavity, orbits, and salivary glands [1]. Deep-seated lesions often occur in the mediastinum and retroperitoneum. Schwannomas are more common in adults and show male predominance [2]. These tumors are almost always solitary and slow-growing, arising from perineural Schwann cells [2]. Primary pleural schwannomas are exceedingly rare. Due to their slow growth and gradual progression, these patients are often asymptomatic, with lesions incidentally discovered [2]. However, large tumors may cause pain or neurological symptoms due to the mass effect and compression of adjacent structures [2-5]. Occasionally, patients may present with pleuritic symptoms, pneumothorax, or hemothorax. The clinical presentation of primary pleural schwannomas often mimics metastatic tumors [1,6,7].

Primary pleural schwannomas account for 1-2% of primary chest wall tumors and are mostly found in the paraspinal thoracic region within the posterior mediastinum. Approximately 5% of these tumors originate from lateral intercostal neuritis arising from autonomic nerve sheaths within the pleural surface [4,8]. Malignant schwannomas are extremely rare, as are the multiple and bilateral pleural schwannomas [9]. Malignancy is typically associated with prior radiation therapy, whereas multiple schwannomas are often seen in neurofibromatosis type II or schwannomatosis [4,5,9]. A pleural mass in an elderly patient, particularly with a history of primary malignancy elsewhere, always raises concern for metastases in the differential diagnosis [1,6].

Diagnosing pleural schwannoma can be extremely challenging, even with advanced imaging modalities. Definitive diagnosis can only be reached by histopathologic examination of biopsy or excision of the tumor. When radiological imaging reveals a solitary, well-defined pleural lesion, schwannoma should be considered in the differential diagnosis, alongside other benign tumors such as lipoma, mesothelioma, and solitary fibrous tumors, among others, as well as malignant tumors, including primary and metastatic lesions. CT scans provide detailed information about the mass, including its size, precise location, encapsulation, and whether the margins are infiltrative or smooth. CT imaging also helps determine if the lesion is solitary or multifocal, as seen in many malignant tumors. Furthermore, CT can reveal the presence of cystic or solid components and heterogeneity, reflecting variable cellularity between Antoni A and Antoni B areas. Typically, Antoni A regions demonstrate contrast uptake, whereas Antoni B areas do not. Although these radiological features are not entirely diagnostic, they are valuable in guiding surgical planning [4,5]. MRI usually shows isointense images on T1-weighted images and hyperintense signals on T2-weighted images, although cystic lesions may have low signal intensity. PET/CT has limited diagnostic utility, as it tends to show high uptake even in benign neoplasms, making it difficult to distinguish these from malignant primary or metastatic tumors [1].

Complete surgical excision is curative for pleural schwannoma. Follow-up imaging can be used in high-risk surgical candidates, the elderly, and occasionally those with small asymptomatic lesions. Awareness of this neoplasm is crucial for clinicians to ensure it is included in the differential diagnosis when assessing intrathoracic, pleural-based lesions.

## CONCLUSION

Herein, we report a rare case of an elderly male patient who presented with severe retrosternal chest pain. The initial chest X-ray revealed a suspicious lesion, which PET-CT confirmed to be left pleural based. Histopathologic examination confirmed this mass to be a benign pleural schwannoma. Despite its extremely rare occurrence by clinicians and radiologists, awareness of this presentation is crucial to ensure accurate diagnosis and timely management.
